# Fascial Manipulation® for chronic aspecific low back pain: a single blinded randomized controlled trial

**DOI:** 10.12688/f1000research.6890.2

**Published:** 2016-01-08

**Authors:** Mirco Branchini, Francesca Lopopolo, Ernesto Andreoli, Ivano Loreti, Aurélie M Marchand, Antonio Stecco

**Affiliations:** 1 Physiotherapy Academic Program, University of Bologna, Bologna, 40138, Italy; 2 Private Practice, Bologna, 40138, Italy; 3 Department of Continuity Assistance and Disability, University of Bologna, Bologna, 40138, Italy; 4 Department of Emergency, University of Bologna, Bologna, 40138, Italy; 5 Private Practice, Padova, 35120, Italy; 6 Sports Medicine Unit, University of Padua, Padova, 35120, Italy

**Keywords:** low back pain, thoracolumbar fascia, myofascial pain, nonspecific pain

## Abstract

Background: The therapeutic approach to chronic aspecific low back pain (CALBP) has to consider the multifactorial aetiology of the disorder. International guidelines do not agree on unequivocal treatment indications. Recommendations for fascial therapy are few and of low level evidence but several studies indicate strong correlations between fascial thickness and low back pain. This study aims at comparing the effectiveness of Fascial Manipulation® associated with a physiotherapy program following guidelines for CALBP compared to a physiotherapy program alone.

Methods: 24 subjects were randomized into two groups, both received eight treatments over 4 weeks. Outcomes were measured at baseline, at the end of therapy and at a 1 month and a 3 months follow-up. Pain was measured with the visual analogue scale (VAS) and the brief pain inventory (BPI), function with the Rolland-Morris disability questionnaire (RMDQ), state of well-being with the short-form 36 health-survey (SF-36). The mean clinical important difference (MCID) was also measured.

Results: Patients receiving Fascial Manipulation® showed statistically and clinically significant improvements at the end of care for all outcomes, in the short (RMDQ, VAS, BPI) and medium term for VAS and BPI compared to manual therapy. The MCID show significant improvements in the means and percentage of subjects in groups in all outcomes post-treatment, in the short and medium term.

Conclusion: Fascial tissues were implicated in the aetiology of CALBP and treatment led to decreased symptomatic, improved functional and perceived well-being outcomes that were of greater amplitude compared to manual therapy alone.

## Introduction

Chronic aspecific chronic low back pain (CALBP) is defined as pain and/or discomfort localised below the costal margin and above the gluteal folds with possible posterior thigh irradiations not extending below the knee; symptoms have to be present for over 3 months or longer than the expected normal healing time ^1^. CALBP is one of the most common and costly syndromes of modern times, with a lifetime prevalence estimated at 70% and reaching 80% in Europe ^2^. Negative impacts of CALBP include for the patient: pain, reduction in activities of daily living, reduced work productivity and/or work absence; for society: increased contacts with health care providers, high demands of medical investigations and related treatments ^3^. Chronicity occurs in 5% of patients suffering from low back pain (LBP) but generates up to 80% of the total costs related to this disorder ^4^.

The multifactorial aetiology of LBP creating pain and functional limitations has been investigated with particular emphasis on physiopathological mechanisms, neuropsychosocial factors and motor control alterations. Several regional, national and international guidelines on LBP treatment exist ^5– 8^ but the levels of evidence supporting these are not always optimal, and a strong recommendation for a specific physiotherapeutic approach is currently lacking. Moreover recent studies have demonstrated an association between thickness and disorganization of the connective tissue (fascia) and CALBP ^9– 11^ but no guidelines could be found regarding the recommendations of a therapeutic approach focused on fascial tissues. For these reasons, it was decided to conduct an experimental study comparing the effectiveness of Fascial Manipulation ^®^ (FM) versus the physiotherapeutic recommendations provided by guidelines (standard manual therapy) (MT).

The null hypothesis was that there would be no difference in short and medium term outcomes between patients undergoing standard physiotherapy only and those where FM was added to standard care.

## Methods

### Study design and patient selection

This study is a single blinded randomized controlled trial aiming at controlling the effectiveness of FM treatment added to a standard protocol of care in patients suffering from CALBP for the primary outcome of pain and secondary outcomes of function and perceived well-being. The study was submitted and approved by the Ethics Committee of “Azienda Ospedaliero-Universitario di Bologna – Policlinico S. Orsola-Malpighi” (n.46/2009/O/Sper, 21/04/2009) and it was conducted according to the principles expressed in the Declaration of Helsinki. Trial was registered in ClinicalTrials.gov with the identifier number NCT01269983. Participants were selected from patients presenting to the outday clinic of Physical Medicine and Rehabilitation Unit of “Azienda Ospedaliero-Universitario di Bologna”, Bologna, Italy. Inclusion criteria were diagnosis of chronic LBP or chronic lumbosciatic pain (above 3 months of duration with daily manifestations), age between 20–60 years old, signed informed consent to the study. Exclusion criteria were neurological signs of spinal stenosis or reflex loss or dysesthesia, continued pharmacological treatment (non-steroidal anti-inflammatory, corticosteroids, painkillers), intake of drugs including antidepressants or anxiolytics or neuroleptics, structural lesions on imaging (spondylolysis or spondylolisthesis, vertebral canal stenosis, secondary vertebral lesions (neoplastic origin), vascular aetiology (i.e. abdominal aneurisms), systemic rheumatological pathologies (i.e. ankylosing spondylitis), comorbidities of the central or peripheral nervous system or cardiovascular or respiratory systems (inability to participate in the therapeutic protocol), insurance claim in act and previous surgery. Patients were screened for inclusion and exclusion criteria by two medical doctors (a physiatrist and an orthopedist). All the participants agreed to avoid any additional therapy or treatment during the study. It was recommended to patients to maintain the posology of their pharmacological therapy. Participants were free to leave the study in case of insurgence of new severe illness.

### Randomization and treatment groups

Patients conforming to both inclusion and exclusion criteria were randomized into a study group (SG) or control group (CG) using a computer generated randomization list. Both groups shared the following treatment characteristics: duration of treatment (4 weeks), frequency (twice a week), duration of treatment session (45 minutes), experience of treating physiotherapists (students of physiotherapy in the third and final year of university training), and each physiotherapist exclusively treated patients belonging to either the SG or the CG. The CG patients followed a program of physiotherapy tailor-made in accordance to the national and international guidelines of CALBP. Treatment consisted of exercises on relaxation, control of diaphragmatic breathing, improved proprioception of the lumbar region, segmental and global stretching of the posterior back and lower limbs musculature, postural re-education, core stability, functional rehabilitation, home exercises. The repetitions and intensity of the manual therapy program was proportional to the patient improving and varied at each session. The SG alternated one treatment of FM and one treatment of MT per week.

FM is a manual therapy that focuses on the deep muscular fascia. This method considers the fascia as a three-dimensional continuum. The mainstay of this manual technique lies in the identification of specific localised areas of the fascia, defined Centre of Coordination (CC) by Luigi Stecco ^12^, where the gliding of the subcutis should be preserved to avoid biomechanical in-coordination of the surrounding muscles. The method is performed by applying a deep friction over the CCs that result more altered at the clinical palpation. The deep friction on these points aims at restoring the physiological gliding properties of the fascia and lead to immediate pain reduction, increased range of motion, improved function that may be objectively evaluated by the therapist ^13, 14^. In FM, the Therapist modulates the treatment in relation to the stiffness/lack of gliding perceived over the CCs, the percentage of pain perceived from the patient and any referred pain the patient may report. The FM Guideline ^12^ indicates manipulating the CCs until the stiffness/lack of gliding have almost disappeared and the patient perceives 60% less pain in comparison to the beginning of the treatment. The referred pain, if any, should also have disappeared.

### Outcome measures

The primary outcome measure of pain was measured using the Visual Analogue Scale (VAS) ^15^ and the sensorial, qualitative and emotional outcome was evaluated with the Brief Pain Inventory (BPI) ^16, 17^, functional outcome was evaluated with the Rolland and Morris Disability Questionnaire (RMDQ) ^18^, quality of life outcome was evaluated with the Short-Form-36-Health Survey (SF-36) ^19^. All of the above were measured at T0 (prior start of therapy). VAS was measured at the start and end of each treatment session whereas BPI, RMDQ and SF-36 were measured at the end of the (T15). The VAS was administered by the physiotherapists pre- and post-treatment in order to evaluate whether a trend was present between FM and MT treatments and between the SG and CG. The RMDQ is composed of 24 functional activities that may be affected by lumbar pain. The SF-36 is a generic psychometric questionnaire evaluating the levels of activity and feelings of well-being, it is composed of 8 scales with multiple questions (36 in total) measuring 8 sections: vitality, physical functioning, bodily pain, general health perceptions, physical role functioning, emotional role functioning, social role functioning and mental health. Scoring ranges from 0 to 100 with higher scores corresponding to higher quality of life.

All outcomes were measured at follow-up at one month (T16) and 3 months (T17) after end of care. The measurements at T0, T15, T16 and T17 were performed by the medical doctor who enrolled the participant in the study who remained blinded to treatment allocation. For the VAS, improvement of 1.5 was considered significant (improvement of or above 4 highly significant), for the BPI the cut-off was 1.5, for the RMDQ the cut-off was improvement of at least 30% ^20^.

### Statistical analysis

Data were encoded into a general database. The software of STATA v10 was used ^21^. Descriptive statistics were used to evaluate differences in outcome measures between groups. Inferential statistics were used to investigate if homogeneity of groups was present at baseline. When interval data was normally distributed Student T-tests were performed, ordinal data were analysed using Mann-Whitney *U* test. The differences in all outcomes at T0, T16, T17 were analysed. RMDQ were analysed as percentage of improvement. Clinical significance was evaluated using the Minimal Clinical Important Difference (MCID) ^22, 23^ for the VAS, RMDQ and BPI.

## Results

Thirty-two patients were recruited from April to December 2009, 24 completed the trial, 6 participants were not enrolled for organizational reasons, 2 withdrew for reasons not directly related to treatment (one had a sport injury, one had a pulmonary infection). From the 24 participants who completed the trial 2 were lost at follow-up (T16, T17). After randomization the SG was composed of 11 patients (4 males, 7 females), the CG was composed of 13 patients (4 males, 9 females). At baseline the VAS was significantly higher in the SG, all other outcomes did not reach statistical significance (see Table 1).

**Table 1.** Baseline values for measured outcomes. S.D. = standard deviation.

Variable at T0	SG	CG
	**Mean (±S.D.)**	**Mean (±S.D.)**
**AGE**	48 (12)	44 (8.2)	**p = 0.374**
**VAS**	5.45 (2.37)	2.62 (1.91)	**z = 0.006**
**BPI**	8.75 (3.86)	7.15 (2.50)	**z = 0.284**
**RMDQ**	6.91 (3.48)	7 (4.04)	**z = 0.977**
**SF36**	57.98 (13.64)	57.71 (16.77)	**z = 0.772**

**Figure 1.**
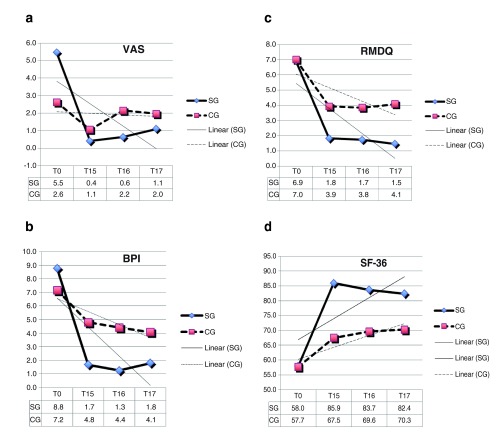
**a**) VAS measures reported per time of evaluation **b**) BPI measures reported per time of evaluation **c**) RMDQ measures reported per time of evaluation **d**) SF-36 measures reported per time of evaluation.

Figure 1a–d shows the results of the measured outcomes at T0, T15, T16 and T17. All outcomes showed improvement for both groups. It can be observed that the improvement is maintained in the medium term (at one and three month follow up) for both groups, however results in the SG were significantly better compared to the CG as seen by the inclination of the trend lines.

Table 2 shows the means, standard deviations, and statistical significance of each outcome for each group. VAS and BPI reached statistical significance for the SG. The outcome of the RMDQ did not reach statistical significance; however the percentage improvement reached significance when measured between T0-T15, T0-T16 a positive trend, not reaching significance, was observed between T0-17 for the group following FM. Table 3 shows the MCID along with the percentage of patients respecting its value for the VAS, RMDQ, BPI for T0-T15, T0-T16, T0-T17. It can be observed that both groups show clinical improvement. Strong clinical significance is reached in the SG for the VAS. In the same group both BPI and RMDQ reached higher clinical significance compared to patients following the program of MT.

**Table 2.** Mean, standard deviation and statistical significance of measured outcomes. S.D. = standard deviation.

VARIABLE		MEAN SG (S.D.)	MEAN CG (S.D.)	Wilcoxon-Mann Whytney [ranksum-test]		
				T0 – T15	T0 – T16	T0 – T17
**VAS**	T0	5.45 (2.37)	2.62 (1.91)	**0.0028**	**0.0010**	**0.0021**
	T15	0.41 (0.54)	1.05 (1.09)			
	T16	0.64 (0.92)	2.15 (1.07)			
	T17	1.09 (1.22)	1.96 (1.27)			
**RMDQ**	T0	6.91 (3.48)	7 (4.04)	**% improvement** **0.0231**	**% improvement** **0.0201**	**% improvement** 0.0868
	T15	1.82 (2.27)	3.92 (3.01)			
	T16	1.73 (2.10)	3.85 (3.05)	0.0849	0.0907	0.0756
	T17	1.45 (2.02)	4.08 (3.95)			
**BPI**	T0	8.75 (3.86)	7.15 (2.50)	**0.0043**	**0.0046**	**0.0147**
	T15	1.68 (1.29)	4.79 (3.18)			
	T16	1.25 (1.79)	4.41 (3.18)			
	T17	1.79 (1.93)	4.07 (3.60)			
**SF36**	T0	57.98 (13.64)	57.71 (16.77)	**0.0057**	0.0642	0.2100
	T15	85.90 (6.47)	67.48 (16.25)			
	T16	83.67 (8.76)	69.62 (18.37)			
	T17	82.39 (8.92)	70.29 (18.21)			

**Table 3.** Clinical significance for measured outcomes as mean differences and percentage of participants.

Difference		VAS		VAS		RMDQ		BPI	
	MCID	1.5		4.0		30%		1.5	
		GS	GC	GS	GC	GS	GC	GS	GC
**T0-T15**	**Mean group difference**	**5.05**	**1.56**	**5.05**	**1.56**	**72.4**	**34.9**	**7.58**	**2.37**
	*% subjects*	*83.3*	*50.0*	*66.7*	*16.7*	*90.9*	*61.5*	*90.0*	*69.2*
**T0-T16**	**Mean group difference**	**4.82**	**0.86**	**4.82**	**0.86**	**74.8**	**36.8**	**8.00**	**2.94**
	*% subjects*	*75.0*	*33.3*	*66.7*	*8.3*	*100.0*	*61.5*	*100.0*	*58.3*
**T0-T17**	**Mean group difference**	**4.36**	**0.65**	**4.36**	**0.65**	**79.5**	**36.2**	**7.46**	**3,27**
	*% subjects*	*83.3*	*33.3*	*50.0*	*8.3*	*100.0*	*53.8*	*100.0*	*58.33*

**Figure 2.**
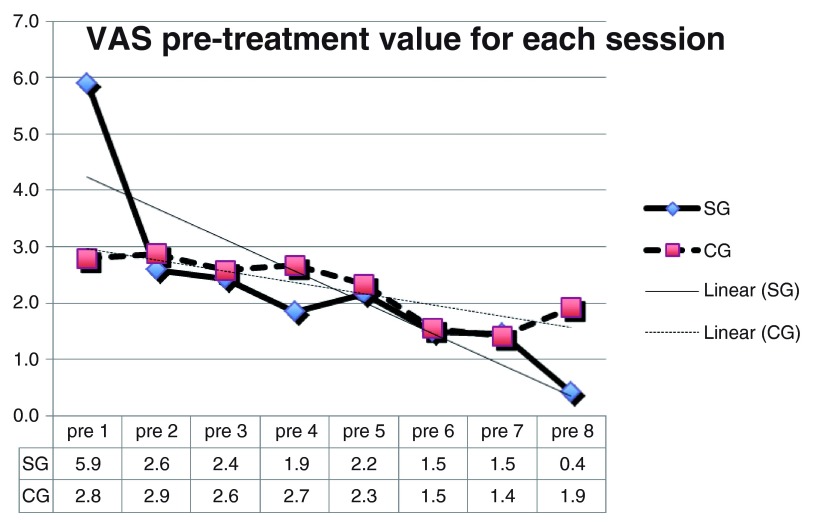
VAS values pre-treatment for each treatment session.

**Figure 3.**
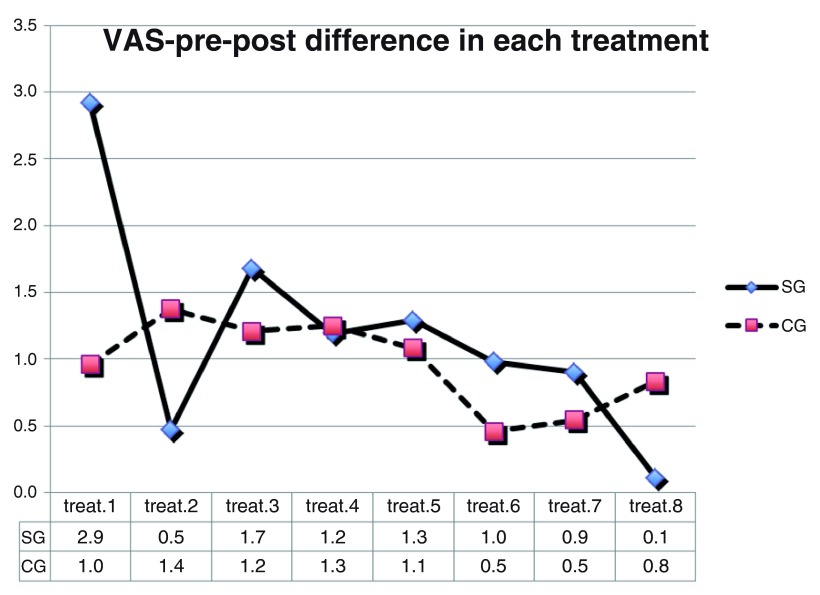
Mean differences in VAS for each treatment sessions.

The values and trend lines of the VAS prior to each treatment are seen in Figure 2. Patients of the SG had a steeper trend, showing higher reduction of pain after each FM treatment. The drop in VAS was statistically significant after the first FM treatment (z=0.0239) for the SG, and reaching significance at the 8 ^th^ manual therapy treatment (z=0.0405) for the CG. The mean difference in VAS value pre- and post-treatment was 2.9 during the first and 1.6 during the third session ( Figure 3). The CG did not reach clinically significant improvement in VAS during treatment sessions. Table 4 shows the mean variation in VAS for the SG was 1.7 for FM treatment and 0.69 for MT treatment. The mean variation in VAS for the CG was 0.96. The trend is more linear for the CG (0.46–1.38 variation) and is steeper for the SG (0.11–2.92 variation).

**Table 4.** Mean VAS variation for each treatment session, mean difference between CG and SG S.D.= standar deviation.

	Session 1 (S.D)	Session 2 (S.D)	Session 3 (S.D)	Session 4 (S.D)	Session 5 (S.D)	Session 6 (S.D)	Session 7 (S.D)	Session 8 (S.D)	Mean MF session	Mean TM session
SG	2.92 (±2.00)	0.47 (±0.60)	1.68 (±2.36)	1.19 (±1.15)	1.29 (±2.00)	0.98 (±1.10)	0.90 (±0.96)	0.11 (±0.21)	1.70	0.69
CG	0.96 (±2.29)	1.38 (±1.87)	1.21 (±1.39)	1.25 (±1.52)	1.08 (±1.61)	0.46 (±1.03)	0.54 (±0.84)	0.83 (±0.83)	//	0.96
z	0.0239	0.1449	0.9733	0.8491	0.8427	0.2977	0.2974	0.0405	

Experimental dataset from Branchini *et al.*, ‘Fascial Manipulation ^®^ for chronic aspecific low back pain: a single blinded randomized controlled trial’ Group 1: Fascial manipulation group 0: Control group. VAS = Visual analogue scale, BPI = Brief pain inventory, RMDQ = Rolland-Morris disability questionnaire, SF-36 = Short-Form 36 health-survey. Click here for additional data file. Copyright: © 2016 Branchini M et al. 2016 Data associated with the article are available under the terms of the Creative Commons Zero "No rights reserved" data waiver (CC0 1.0 Public domain dedication).

## Discussion

FM treatment sessions, associated to four treatments of MT over 4 weeks in chronic aspecific low back pain, resulted in significant statistical and clinical improvement compared to eight treatments of MT alone at end of therapeutic intervention, at 1 month and at 3 months follow up. Therefore the null hypothesis was rejected.

This is the first randomized trial evaluating the effectiveness of FM for CALBP. The results observed in the MT group are similar to those reported in the literature ^24, 25^.

With regards to methodology it was decided to apply the exclusion criteria inherent to diagnostic criteria proposed in the specific guidelines for low back pain. These recommend a diagnostic triage of patients to exclude specific spinal pathologies or pain caused by compression on nerve roots. The outcome measure of pain was evaluated with the VAS and the BPI. The BPI was chosen as outcome measure because it is a complete and multidimensional test that evaluates the sensorial, qualitative and emotional aspects of pain. It initially started as a scale for oncological patients but its validity has also been proven in patients with chronic pain of non-neoplastic origin ^17^. The SF-36, even though it is not specific for low back pain, was chosen as an outcome for perceived well-being.

Both groups were homogeneous at baseline except for the VAS value. Both followed a course of eight treatment sessions performed twice a week over 4 weeks. In both groups the therapists had the same level of education. Improvement in all outcomes was observed for both groups with the SG showing greater statistically and clinically significant improvements. The initial VAS value was higher for the SG which may have skewed the results towards a better improvement for the SG. However it has to be noted that both the VAS and the BPI reached statistical significance at all measurement times for the SG. This indicates that the FM treatment also created a significant improvement in another outcome that was similar at baseline. Therefore it may be considered that the VAS improvement reported in the SG is not solely related to the higher VAS at baseline.

The VAS values pre- and post- each treatment allow us to evaluate which intervention (MT or FM) showed the best improvement in the SG. It can be observed that the values post-FM treatment showed greater diminution compared to the CG and compared to the MT treatment performed in the SG. Indeed the first and third treatments in the SG showed a greater difference pre- and post-treatment. It can be observed that the first FM treatment lead to a statistically significant decrease in VAS compared to the CG. Such results can support the rationality of the FM method that, restoring the sliding of the fascia proximally and distally from the area of the pain, is able to recover the range of motion, decrease the stiffness and painful sensation perceived by the patient. With regards to the VAS improvement in the SG, it may be argued that, at the 8 ^th^ treatment, statistically significant improvement would be more difficult to reach with a VAS value pre-treatment of 0.4, compared to 1.9 in the CG.

The perceived state of well-being showed significant improvement at the end of therapeutic intervention, in the FM and MT group, compared to the MT group only. At follow-up the values did not reach statistical significance, but showed a better trend for the SG. Functionality (of the RMDQ scale) reached statistical significance at the 1 month follow-up.

Clinical significance showed higher mean of improvement for all outcomes for the SG. Also the percentage of patients having higher values of MCID was higher than the CG and reaching 100% for BPI and RMDQ at T16 and T17. This indicates that FM treatment, when added to MT treatment, leads to clinically significant improvement in the short and medium term with regards to severity of pain and disability.

The rationale for including FM treatment for patients suffering from CALBP is based on the fact that fascia is a type of dense connective tissue on which about 30% of muscle fibres have either insertions or origins ^26^. Fascia is therefore tensioned during any kind muscular activity in any direction of movement; it transmits tensions towards the perimysium and towards synergic muscular groups ^27, 28^. The deep fascia is composed of layers of dense connective tissue that are dedicated to transmitting the load and loose connective tissue that allows the gliding of the collagen layers. Different authors have demonstrated the rich innervation of the fascial tissue and in particular of the thoracolumbar fascia ^29– 32^. Langevin ^9, 33^ has demonstrated an increase of the thickness of the thoraco-lumbar fascia and a decrease of the gliding of the different layers. It is hypothesised that the layers of loose connective tissue are the ones causing the increase in thickness because fibrosis is not recognized in subject with CALBP. Recent studies have demonstrated that the major component of the loose connective tissue, the hyaluronan, by increasing concentration and/or size, begins to entangle into complex arrays, leading to a decrease in the gliding properties of the fascia ^34^. Due to the viscoelastic properties of the loose connective tissue, allowing it to modulate the dynamic response of the mechanoreceptor, we hypothesize that a stiff thoracolumbar fascia can alter the receptor afferents ^35, 36^ providing another mechanism to the multifactorial aetiology of chronic back pain ^37^.

The FM treatment utilises the non-Newtonian properties of the hyaluronan, by applying continuous shear to the fascial tissue, to increase the temperature of the fascial tissue. This leads to the destruction of the van der Waals and hydrophobic forces that hold the hyaluronan chains together. A temperature of 40°C is able to decrease the viscosity of the hyaluronan ^38^ and increases its ability to glide ^39^. A less viscous loose connective tissue allows fibroblasts to perceive the lines of tensions of the fascial layers and may therefore lead to the remodelling of their dense connective tissue with a deposition of collagen fibres in the correct lines of force ^40^.

### Limitations

This study demonstrates that FM + MT reached clinical and statistical significance in outcomes compared to MT alone. However the sample size was small because, to avoid a long recruitment period that could generate bias in the data evaluation, it was difficult to increase the number of subjects treated in each group. Due to the small size and the short follow up, we suggest considering this data as preliminary findings. Validated self-administered scales do not investigate variations in anatomical findings such as thickness of connective tissue as highlighted with diagnostic imaging including ultrasounds ^5^. It was therefore not possible to evaluate whether pain changes reported by patients were related to anatomical changes in this study. Further, the discrepancy in baseline VAS score may have affected the statistical evaluation, but it has to be noted that, in the SG, both the VAS and the BPI reached statistical significance at all measurement times, indicating that FM treatment created significant improvement in another outcome that was similar at baseline. For this reason, in SG, the VAS improvement cannot be solely related to the higher VAS at baseline. Statistical correction for the different VAS value at baseline could be considered.

## Conclusions

This study shows that the implementation of FM, within a course of MT following established guidelines, reaches statistically and clinically significant improvement in the outcomes of pain, function and quality of life in patients suffering CALBP both at the end of care as at one and three months follow-up compared to MT alone. Considering the costs of CALBP to health care systems it may be considered to implement FM treatment as part of routine care in existing physiotherapy programs. Considering the significant decrease in pain especially following the first two FM treatment sessions, it may be hypothesized that FM may reduce the number of treatments required for patients with CALBP and therefore reduced the overall costs to health care systems and patients.

## Data availability

The data referenced by this article are under copyright with the following copyright statement: Copyright: © 2016 Branchini M et al.

Data associated with the article are available under the terms of the Creative Commons Zero "No rights reserved" data waiver (CC0 1.0 Public domain dedication). 

*F1000Research*: Dataset 1. Experimental dataset from Branchini *et al.*, ‘Fascial Manipulation ^®^ for chronic aspecific low back pain: a single blinded randomized controlled trial’, 10.5256/f1000research.6890.d100559 ^41^

## Consent

Written informed consent for publication of clinical details was obtained from the patients.
