# Supplementary material for: Fascial Manipulation® for chronic aspecific low back pain: a single blinded randomized controlled trial
Source: F1000Res. 2016 Jan 8;4:1208. Originally published 2015 Nov 3. [Version 2] doi: 10.12688/f1000research.6890.2 (PMC4706049; doi:10.12688/f1000research.6890.2)
Supplement: Peer review history

# Peer review: Referee response for version 1

**DOI:** 10.5256/f1000research.7417.r11667

**Published:** 2015-12-21

**Authors:** Heidi Prather

**Affiliations:** 1 Division of Physical Medicine and Rehabilitation, Department of Orthopaedic Surgery, School of Medicine, Washington University, St. Louis, MO, USA

Well written manuscript regarding a controversial yet very important topic: the management of axial low back pain.

Comments:

1. I am assuming people with a history of surgery were excluded. Is this an appropriate assumption?
2. How long had these people experienced low back pain? Did they have pain every day or exacerbations?
3. Myofascial manipulation requires the treating provider to be experienced with the evaluation of the patient that then directs treatment. How did you attempt to standardize this?

I have read this submission. I believe that I have an appropriate level of expertise to confirm that it is of an acceptable scientific standard.

# Peer review: Referee response for version 1

**DOI:** 10.5256/f1000research.7417.r11477

**Published:** 2015-12-09

**Authors:** Olavi Airaksinen

**Affiliations:** 1 Department of Physical and Rehabilitation Medicine, Kuopio University Hospital, Kuopio, Finland

Nice article. In general format it looks very good. The presentation style is clear – good and informative figures.

The critical points that the article and the researchers have realized  is the limitations of a sample size 24 to divided two groups – so 12 per group. The other critical issue will be the follow up time up to 3 months. So on that background I prefer to talk with these results as preliminary findings – which were very promising and will stimulate to work more with this and plan next trial with bigger population and longer follow up - up to one or two years (taking count that the patients were chronic LBP patients).

Or one possibility will be add the phrase 'a pilot study' to the title?

We have to be careful when making conclusions based on preliminary and pilot studies – the results seem to be promising – and I like to say that this way.

As a conclusion I would prefer the indexation of this article with my suggested minor revisions.

I have read this submission. I believe that I have an appropriate level of expertise to confirm that it is of an acceptable scientific standard.
